# Targeting Glutamate Excitotoxicity With Memantine Modulates Glial Response and Protects Motoneurons After Spinal Root Lesion

**DOI:** 10.1111/jnc.70429

**Published:** 2026-04-08

**Authors:** Arthur Ventura Martins Leão, Gabriel Gaspar Bíscaro, Alexandre Leite Rodrigues de Oliveira, Luciana Politti Cartarozzi

**Affiliations:** ^1^ Laboratory of Nerve Regeneration Institute of Biology—University of Campinas Campinas Brazil

## Abstract

Spinal root injuries trigger longitudinal spinal cord damage, leading to motoneuron degeneration, gliosis, and synaptic loss. Glutamate excitotoxicity through NMDA and AMPA receptor overstimulation is a key driver of this pathology, highlighting NMDA receptor antagonists as potential neuroprotective agents. Here, we evaluated the effects of memantine after unilateral L4–L6 ventral root crush (VRC) in adult C57BL/6JUnib mice. Animals received daily oral gavage of vehicle or memantine (30, 45, or 60 mg/kg) for 14 days. At 28 days post‐injury, histological analysis showed that memantine reduced astrogliosis and microglial activation, while enhancing motoneuron survival (most pronounced at 45 mg/kg, *p* < 0.001) and preserving synaptic coverage (*p* < 0.01), without significant changes in VGLUT‐1 or GAD65 expression. Consistently, RT‐qPCR analysis revealed early upregulation of inflammatory markers (*Ccr2, Itgam*) in vehicle‐treated mice, which was attenuated by memantine at 3–7 days post‐injury (*p* < 0.05). These findings indicate that memantine confers neuroprotection in VRC by modulating inflammatory gene expression, mitigating gliosis, and promoting motoneuron survival, supporting its therapeutic potential in spinal cord injuries.

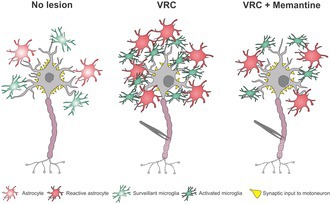

## Graphical abstract

Motoneuron axotomy induced marked neuroinflammation, synaptic withdrawal, and structural remodeling in the ventral root crush (VRC) model, characterized by intense microglial and astroglial activation and reduced synaptic coverage. Memantine treatment produced consistent, dose‐dependent neuroprotection, with maximal effects at 45 mg/kg. Despite persistent axonal injury, memantine attenuated glial reactivity, suppressed early pro‐inflammatory gene expression, and preserved synaptic apposition onto injured motoneurons. These effects resulted in improved maintenance of inhibitory–excitatory balance and spinal synaptic organization, supporting sustained neuronal survival during the post‐lesion remodeling phase.

**Abbreviations**
AMPA: α‐Amino‐3‐hydroxy‐5‐methyl‐4‐isoxazolepropionic acid receptor
ANOVA: analysis of variance
ATP: adenosine triphosphate
Ccr2: C‐C chemokine receptor type 2
cDNA: complementary DNA
CL: contralateral
CNS: central nervous system
DAPI: 4′,6‐diamidino‐2‐phenylindole (nuclear stain)
DNA: deoxyribonucleic acid
GAD65: glutamate decarboxylase 65
GFAP: glial fibrillary acidic protein
GLAST: glutamate aspartate transporter
Glt1: glutamate transporter 1 (gene symbol, also EAAT2)
Glul: glutamine synthetase (gene symbol)
Gria2: glutamate ionotropic receptor AMPA type subunit 2 (gene symbol)
Iba‐1: ionized calcium‐binding adapter molecule 1
IL: ipsilateral
Il‐1β: interleukin 1 beta
Il‐6: interleukin 6
Itgam: integrin alpha M (gene symbol, also CD11b)
MHC‐I: major histocompatibility complex I
NMDA: N‐Methyl‐d‐Aspartate Receptor
O.C.T.: optimal cutting temperature compound
PB: phosphate buffer
PBS: phosphate‐buffered saline
PCR: polymerase chain reaction
qPCR: quantitative polymerase chain reaction
RGB: red, green, blue
ROI: region of interest
RRID: research resource identifier
Tgf‐β: transforming growth factor beta
Tnf‐α: tumor necrosis factor alpha
VGLUT‐1: vesicular glutamate transporter 1
VRC: ventral root crush

## 1 Introduction

Injuries affecting the spinal roots are a common form of proximal peripheral nerve damage, often resulting in significant functional impairment. Such lesions not only affect the peripheral nervous system but also induce retrograde longitudinal alterations in the spinal cord, such as motoneuron loss, reactive gliosis, and synaptic stripping, which compromise motor function and neural plasticity, posing a major clinical challenge (Carlstedt 2007).

Proximal axotomy reduces excitatory and inhibitory inputs onto motoneurons (synaptic stripping), with disproportionate loss of excitatory terminals and maladaptive remodeling of anterior horn circuitry (Alvarez et al. 2010, 2020; Linda et al. 2000; Oliveira et al. 2004). In particular, after a peripheral nerve lesion, Ia proprioceptive afferents labeled by VGLUT‐1 undergo persistent retraction of synaptic varicosities on motoneurons, contributing to loss of the stretch reflex despite possible muscle reinnervation (Alvarez et al. 2011; Rotterman et al. 2019, 2014).

Neuroinflammation critically shapes these processes. Microglial activation and recruitment of CCR2‐positive macrophages around injured motoneurons destabilize synapses and may hinder recovery (Rotterman et al. 2019). Recent transcriptomic studies further show dynamic glial and immune responses in the early post‐injury phase, with increased vascular permeability, cytokine release, and immune cell infiltration, followed by astroglial scar formation and persistent microglial alterations (Li et al. 2022). These findings identify microglia and astrocytes as key targets for interventions aimed at preserving motoneuron survival.

Importantly, these pathological processes unfold in a defined temporal sequence. Seven days after the lesion, an early microglial reaction coupled with modest motoneuron loss and a significant reduction in the synaptic coverage are evident. By 14 days, motoneuron loss and synaptic stripping become more intense. By 28 days, strong astrogliosis, sustained motoneuron loss and synaptic stripping are well established (Cartarozzi et al. 2025, 2022, 2019). This pattern indicates a cumulative cascade of secondary injury mechanisms. Thus, the first 2 weeks after injury are a particularly sensitive window for therapeutic intervention, before the glial remodeling, large synaptic detachment and motoneuron death are consolidated.

Within this context, glutamate excitotoxicity emerges as a major contributor to progressive motoneuron loss. NMDA receptor overactivation leads to excessive Ca^2+^ influx and downstream pathological cascades including mitochondrial dysfunction, ATP depletion, and caspase activation, ultimately culminating in neuronal death (Lau and Tymianski 2010). Pharmacological antagonism of NMDA receptors has been explored to mitigate these effects. While several compounds showed preclinical neuroprotection, adverse effects have limited clinical application (Lau and Tymianski 2010).

Memantine, a low‐affinity, non‐competitive NMDA receptor antagonist, has a favorable pharmacological profile due to its weak, reversible interaction with the NMDA channel pore and high dissociation rate, allowing it to block pathological but not physiological activity (Alam et al. 2017; Song et al. 2018). In addition, evidence indicates that memantine preferentially inhibits extrasynaptic over synaptic NMDAs (Kuns et al. 2025; Xia et al. 2010), a property that contributes to its ability to limit excitotoxic signaling while preserving normal synaptic transmission.

Here, we evaluated the neuroprotective potential of memantine following ventral root crush in mice, with emphasis on motoneuron survival, microglial and astroglial responses, synaptic coverage, and the expression of inflammatory and glutamatergic genes.

## 2 Materials and Methods

### 2.1 Animals and Experimental Groups

Female C57BL/6JUnib mice (aged 6 to 8 weeks, weighing approximately 20 g) were obtained from the Multidisciplinary Center for Biological Research (CEMIB) at UNICAMP. Animals were housed in the Laboratory of Nerve Regeneration (Institute of Biology, University of Campinas), with a maximum of 5 mice per cage. Polysufone cages had filter lids, stainless steel dividers, with irradiated wood shavings as bedding. Animals had *ad libitum* access to filtered water and irradiated industrial chow. Environmental conditions were maintained under a 12‐h light/dark cycle, a temperature of approximately 23°C, and 60% humidity.

Experimental groups were defined based on treatment conditions, survival period, and surgical procedure employed (Table 1). Animals were arbitrarily assigned to the experimental groups and labeled with unique numerical codes to ensure unbiased data acquisition and analysis. The numerical coding was performed by Experimenter A, who conducted the surgeries and administered the treatments and therefore was aware of group allocation. Experimenter B, responsible for image acquisition and quantitative analysis, remained fully blinded to treatment identity throughout the entire analysis workflow. The numerical code was only revealed after all imaging and quantification were completed.

**TABLE 1:** Experimental groups distributed by technique and survival time.

Experimental group	3 dpi	7 dpi	28 dpi		
	RT‐qPCR		Immunohistochemistry (anti‐GFAP, anti‐Iba‐1, anti‐Synaptophysin)	Immunohistochemistry (anti‐VGLUT‐1, anti‐GAD65)	Motoneuronsurvival
Memantine 30 mg/kg	—	—	5	5	5
Memantine 45 mg/kg	5	4	5	5	5
Memantine 60 mg/kg	—	—	5	5	5
Vehicle	5	5	5	5	5
Control (no lesion)	6		—	—	—

All experimental procedures were conducted in accordance with the Brazilian College of Animal experimentation guidelines, which were approved by the Institutional Committee for Ethics in Animal Experimentation (CEUA/IB/UNICAMP, protocol numbers: 5740‐1/2021, 5740‐1(A)/2022, and 6428‐1/2024).

The sample size was estimated using the formula for comparing two means with equal variances in a two‐tailed test: $n=\frac{2s^{2}\left(Z_{1-\frac{\alpha }{2}}+Z_{1-\beta }\right)2}{∆2}$. Using *s* = 0.2, *α* = 0.05, $Z_{1-\frac{\alpha }{2}}=1.96$, 80% power ($Z_{1-\beta }=0.84$) and a minimum detectable difference Δ ≈ 0.43, the formula yielded *n* = 3.36. This was rounded up to 4 animals per group, with 5 animals allocated to account for potential experimental losses.

### 2.2 Surgical Procedure—Ventral Root Crush Injury

Mice were anesthetized with intraperitoneal injections of Xylazine (Anasedan, Ceva, Brazil; 10 mg/kg) and Ketamine (Dopalen, Ceva, Brazil; 100 mg/kg). To prevent corneal desiccation, ophthalmic ointment containing dexpanthenol (Epitegel, Bausch + Lomb, Germany) was applied. Animals were placed on a thermostatically controlled heating pad and maintained under inhalational anesthesia with isoflurane (1.0%–1.5% in medical air). Following dorsal trichotomy and confirmation of complete loss of the paw withdrawal reflex, a longitudinal skin incision was performed parallel to the vertebral column at the thoracolumbar junction. Paravertebral musculature was carefully retracted to expose the vertebral laminae.

**FIGURE 1:**
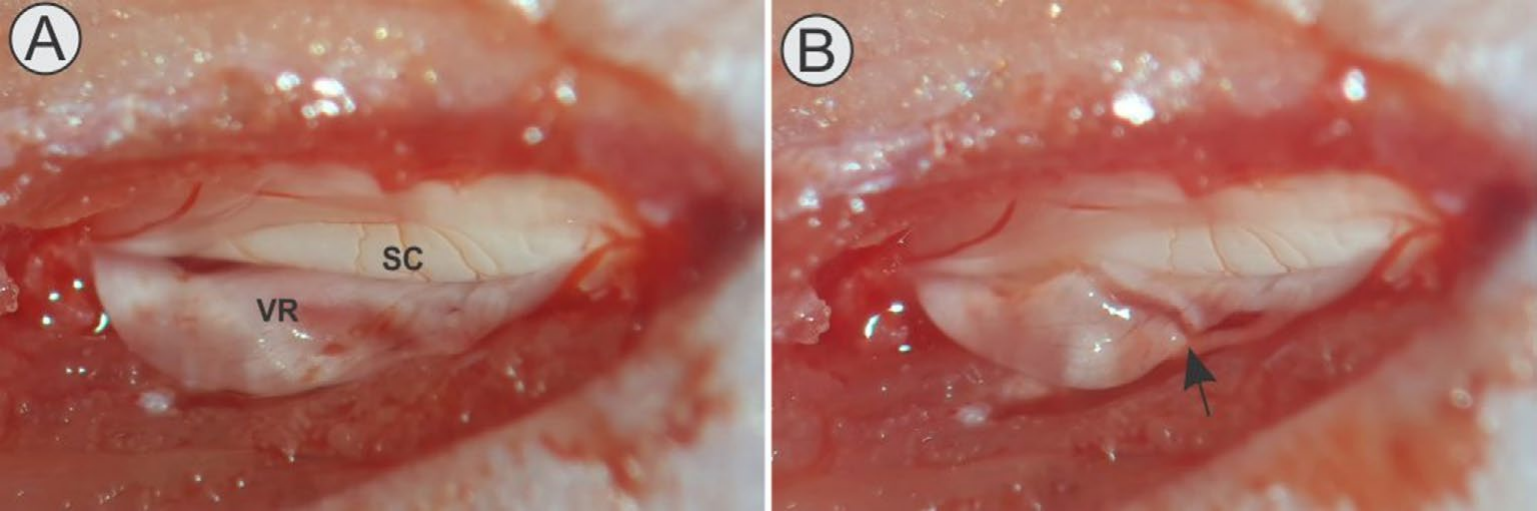
Surgical filed view of the ventral root crush (VRC) process, showing in (A) the spinal cord (SC) and the set of ventral roots L4, L5 and L6 (VR) after the dura‐mater opening, and later, in (B) the site of crushing of the ventral roots, identified by the black arrow.

A unilateral laminectomy was then conducted to access the lumbar enlargement of the spinal cord. Subsequently, the dura mater was incised to expose the ventral roots corresponding to spinal segments L4, L5, and L6 (Figure 1). Ventral root crush was performed by applying three consecutive compressions, each lasting 10 s, using a n°. 4 forceps. Following the injury, muscle and skin were sutured in anatomical layers. Animals were maintained under thermal support until full recovery from anesthesia.

Postoperative analgesia consisted of subcutaneous administration of Tramadol (Neoquímica, Brazil; 5 mg/kg) immediately after surgery and subsequently once daily for three consecutive days. Surgical success was routinely verified by assessing motor behavior over the days following injury. Mice exhibited complete paralysis of the ipsilateral hindlimb as a reliable indicator of effective ventral root crush. Additionally, histological examination of spinal cord cross‐sections consistently revealed morphological signs of root degeneration, further confirming the integrity of the lesion. In the present study, all the animals met the inclusion criteria, and no exclusions were made.

### 2.3 Drug Preparation and Administration Protocol

Memantine was prepared from commercially available tablets (Maizher, Prati, Donaduzzi & Cia, Brazil) by maceration. According to the manufacturer's certificate, each tablet contains 8.31 mg of the active compound (memantine hydrochloride). The number of tablets was calculated to obtain a final solution concentration suitable for oral gavage in mice, using water as the vehicle. All experimental groups received memantine at a fixed concentration of 3 mg/mL. The volume administered was individually adjusted based on the animal's body weight, the solution concentration, and the target dose, according to the following formula:

$$\begin{array}{c} \text{Volume}\;\left(\mathrm{mL}\right)=\text{Dose}\left(\frac{\mathrm{mg}}{\mathrm{kg}}\right)\times \frac{\text{body weight}\;\left(g\right)}{1000\left(\frac{g}{\mathrm{kg}}\right)}\\ \;\times \frac{1}{\text{solution concentration}\;\left(\frac{\mathrm{mg}}{\mathrm{ml}}\right)} \end{array}$$

Drug or vehicle was administered via oral gavage once daily, starting 30 min after surgery, for up to 14 days depending on the experimental group. For animals with a 28‐day survival period, treatment was performed for the first 14 days post‐injury, followed by an additional 14 days without treatment prior to euthanasia.

### 2.4 Perfusion and Tissue Processing

Mice were deeply anesthetized with an overdose of ketamine (300 mg/kg) and xylazine (30 mg/kg) and underwent thoracotomy followed by transcardial perfusion with phosphate‐buffered saline (PBS; 0.1 M sodium phosphate buffer with 0.9% NaCl; pH 7.38), followed by 4% paraformaldehyde (Sigma‐Aldrich, cat. n°.: P6148) in 0.1 M phosphate buffer (PB) as fixative. Lumbar spinal cord enlargements were dissected and post‐fixed overnight (12 h) at 4°C in the same fixative. Subsequently, tissues were rinsed in 0.1 M PB and cryoprotected in ascending sucrose solutions (10%, 20%, and 30%, 12 h each at 4°C). For embedding, samples were placed in Tissue‐Plus O.C.T. compound (Fisher HealthCare, cat. n°.: 23‐730‐571), frozen in n‐hexane (Dinamica, cat. n°.: P1005820030783) cooled to −32°C to −35°C and stored at −22°C until sectioning. Transverse spinal cord sections (12 μm thick) were obtained using a cryostat (Microm HM525), mounted on gelatin‐coated slides, and stored at −22°C until use.

### 2.5 Neuronal Survival

Transverse sections of the lumbar enlargement were stained with toluidine blue (Synth, cat. n°.: A1111.01.AD) to perform motoneuron quantification. Neurons were counted in every third section, totaling approximately 15 sections per animal. For the staining protocol, slides were left at room temperature for 30 min, subsequently immersed in 0.05% toluidine blue solution (prepared in distilled water) for 30 s, rinsed in distilled water, dehydrated, cleared, and mounted using Permount (Fisher Scientific, cat. n°.: SP15‐500) and coverslips.

Histological analyses were performed under the light microscope Leica DM5500B equipped with a DFC295 camera. Motoneurons were identified within the dorsolateral motoneuron pool of lamina IX of Rexed (Figure 1A,B), delineated as the established region containing motoneurons that supply the distal hindlimb muscles (Bacskai et al. 2014). Quantification was performed bilaterally (ipsilateral injured side and contralateral uninjured side). Only neurons exhibiting a clearly visible nucleus were counted. This standard criterion prevents double counting across serial sections and ensures that each neuron is sampled at its maximal somatic diameter. Because neurons lacking a visible nucleus do not constitute independent countable profiles within this stereological approach, no separate record of “excluded cells” is generated.

To prevent overestimation due to split‐cell counting, the Abercrombie correction was applied (Abercrombie and Johnson 1946): *N* = (*n* · *t*)/(*t* + *d*), where *N* = corrected number of neurons, *n* = raw count, *t* = section thickness (48 μm), and *d* = mean neuronal diameter. For comparative analysis, ipsilateral/contralateral ratios were computed per animal and averaged for group comparisons.

### 2.6 Immunohistochemistry

Slides were acclimatized to room temperature, rinsed in phosphate buffer (PB 0.01 M), and incubated with 3% bovine serum albumin (BSA, Sigma‐Aldrich, cat. n°.: A3912) in PB for 45 min to block nonspecific binding. Primary antibodies (anti‐GFAP, Abcam, ab7260, 1:750—RRID:AB_305808; anti‐Iba‐1, Wako, 019‐19741, 1:750—RRID:AB_839504; anti‐synaptophysin, Novus Biologicals, NBP2‐25170, 1:1000—RRID:AB_2814699; anti‐VGLUT‐1, Synaptic Systems, 135303, 1:1000—RRID:AB_887875, and anti‐GAD65, Abcam, ab26113, 1:750—RRID:AB_448989) were incubated *overnight* in humidified chambers. After rinsing (3 × 5 min with PB 0.01 M), slides were incubated for 1 h at room temperature with fluorophore‐conjugated secondary antibodies (Alexa488 goat‐anti‐rabbit, Jackson ImmunoResearch, 111.545.008, 1:500—RRID:AB_2338048; Cy2 donkey‐anti‐mouse, Jackson ImmunoResearch, 715.225.150, 1:500—RRID:AB_2340826) and DAPI (ThermoFisher, cat. n°.: D1306, 1:1000). Finally, slides were rinsed again and mounted with a glycerol/PB 0.1 M solution (3:1).

### 2.7 Imaging Acquisition

Immunostained sections were observed under a fluorescence microscope (Leica DM5500B) and documented with a digital camera (Leica DFC345FX), using the specific filters according to the secondary antibodies or DAPI, at the LAS X software (3.6.0.20104; Leica).

For imaging acquisition, a series of standardizations were made in terms of exposure time and camera gain for each antibody. All sets of images destined for the integrated density of pixels quantification were acquired with the 20× objective, using the same acquisition parameters in a single focal plane (2D), both at the contralateral and ipsilateral sides of the dorsolateral motor nuclei within the ventral horn of the spinal cord in three sections per animal.

For the morphological analysis of microglia, maximum intensity projections were obtained from an 11.95 μm *z*‐stack (with a *z*‐step size of 1.49 μm) followed by deconvolution of the dorsolateral nucleus of Rexed lamina IX from Iba‐1 immunolabeling. All image sets were acquired using the same software parameters. For quantification, two images from each animal were used.

### 2.8 Integrated Density of Pixels Quantification

The integrated density of pixels was used to quantify the intensity of immunolabeling for all the markers with ImageJ software (version 1.33u, National Institutes of Health, USA). Initially, 8‐bit images were segmented using the threshold tool. The threshold value was defined by comparing it to the corresponding RGB image. For GFAP and Iba‐1, the entire image was quantified. For synaptophysin, eight circular regions of interest (ROIs) were positioned equidistantly around two sampled motoneurons. For VGLUT‐1 and GAD65, one circular ROI covering the dorsolateral motor nucleus was quantified (Figure S1). After the quantification, a mean was calculated for each animal, followed by the mean and standard error of the mean for each experimental group.

### 2.9 Microglial Morphological Analysis

To investigate the morphological changes that occur in microglia after axotomy, the maximum intensity projections of Iba‐1 immunolabeling were used. Iba‐1‐positive cells showing visible soma, and discernible projections were classified into five types according to the criteria: Type I: small cells with 1 or 2 processes; Type II: cells with 3–5 short processes; Type III: cells with more than 5 long, thick processes with small soma; Type IV: cells with enlarged soma and retracted, thick processes; Type V: ameboid soma with multiple short processes (Cartarozzi et al. 2022; Diz‐Chaves et al. 2012; Lopez‐Rodriguez et al. 2015).

The proportion of each microglial type was calculated relative to the total number of classified cells per image, with means computed for each animal and group. For analysis, types I and II were considered “surveillant” microglia, while types III, IV, and V were categorized as “activated” microglia.

### 2.10 Gene Expression Analysis—RT‐qPCR

For RT‐qPCR analysis, tissue collection was performed at 3 and 7 dpi, time‐points selected to capture the early molecular events associated with the acute inflammatory response and glial activation that follow motoneuron axotomy. At each predetermined time‐point, mice were euthanized via overdose of ketamine and xylazine, followed by transcardial perfusion with phosphate‐buffered saline (0.9% NaCl in 0.1 M sodium phosphate buffer, pH 7.38). The lumbar enlargements were then carefully dissected, separated into ipsilateral and contralateral segments, placed in pre‐labeled 1.5 mL microtubes, snap‐frozen in liquid nitrogen, and stored at −80°C until further processing. Further analysis was conducted on the ipsilateral sides of the spinal cord.

Total RNA was extracted using TRIzol Lysis Reagent (ThermoFisher, Cat. No. 15596018) according to the manufacturer's instructions. RNA concentration and purity were assessed using a nanophotometer (Implen P330). Although 5 mice per group were targeted, one sample from the memantine 45 mg/kg (7 dpi) group failed to yield sufficient RNA, resulting in *n* = 4 for that group. Complementary DNA (cDNA) synthesis was performed using the High‐Capacity cDNA Reverse Transcription Kit (Applied Biosystems, Cat. No. 4368814), following the manufacturer's protocol. Each reaction used 1.0 μg of total RNA as input. The synthesized cDNA was used as the template for quantitative PCR (qPCR) reactions, carried out in triplicate. Reactions were prepared with TaqMan Gene Expression Master Mix (2×) (Life Technologies, cat. n°.: 4369016), RNase‐free water, and TaqMan Gene Expression Assays (including primers and hydrolysis probes) specific for the target genes listed in Table 2, in a total volume of 20 μL per reaction.

**TABLE 2:** TaqMan assays used in qRT‐PCR analyses.

Gene	Assay ID
*Glast (Slc1a3)*	Mm00600697_m1
*Glul (Glutamine synthetase)*	Mm00725701_s1
*Gria2 (GluA2)*	Mm00442822_m1
*Ccr2*	Mm99999051_gH
*Itgam (CD11b)*	Mm00434455_m1
*Glt1 (Slc1a2)*	Mm01275814_m1
*Tnf‐α*	Mm00443258_m1
*Tgf‐β*	Mm01178820_m1
*Il‐1β*	Mm00434228_m1
*Il‐6*	Mm00446190_m1

PCR reactions were run under the following conditions: an initial denaturation at 95°C for 10 min, followed by 45 cycles of 95°C for 15 s and 60°C for 1 min for annealing/extension. *Hprt1* (Mm01545399_m1) was chosen as the reference gene due to its consistent expression across all experimental groups. Amplifications were carried out on the MX3005P system (Agilent, Santa Clara, CA, USA), with data acquisition and processing performed using MxPro software (Agilent). For statistical evaluation, the average of each sample's triplicates was considered as one biological replicate. Relative gene expression levels were calculated according to the 2^−ΔΔCt^ method (Livak and Schmittgen 2001).

### 2.11 Statistical Analysis

Quantitative data were analyzed using GraphPad Prism version 8.0 (GraphPad Software, San Diego, CA, USA). For each animal (*n* = 4–6 per group) mean values were calculated from multiple technical replicates (multiple images analyzed for histological quantifications or triplicate RT‐qPCR measurements). The animal means were then compared across groups using a one‐way analysis of variance (ANOVA), followed by Tukey's post hoc test for multiple comparisons. Data normality was assessed using the Shapiro–Wilk test, and all datasets used in parametric analyses met the assumption of normal distribution (*p* > 0.05). Two‐way ANOVA followed by Tukey's multiple comparisons test was performed to analyze the microglial cell types. The statistical significance thresholds were set at *p* < 0.05 (*), *p* < 0.01 (**), and *p* < 0.001 (***). All data are expressed as mean ± standard error of the mean (SEM).

## 3 Results

### 3.1 Effect of Memantine Treatment on Spinal Motoneuron Survival

**FIGURE 2:**
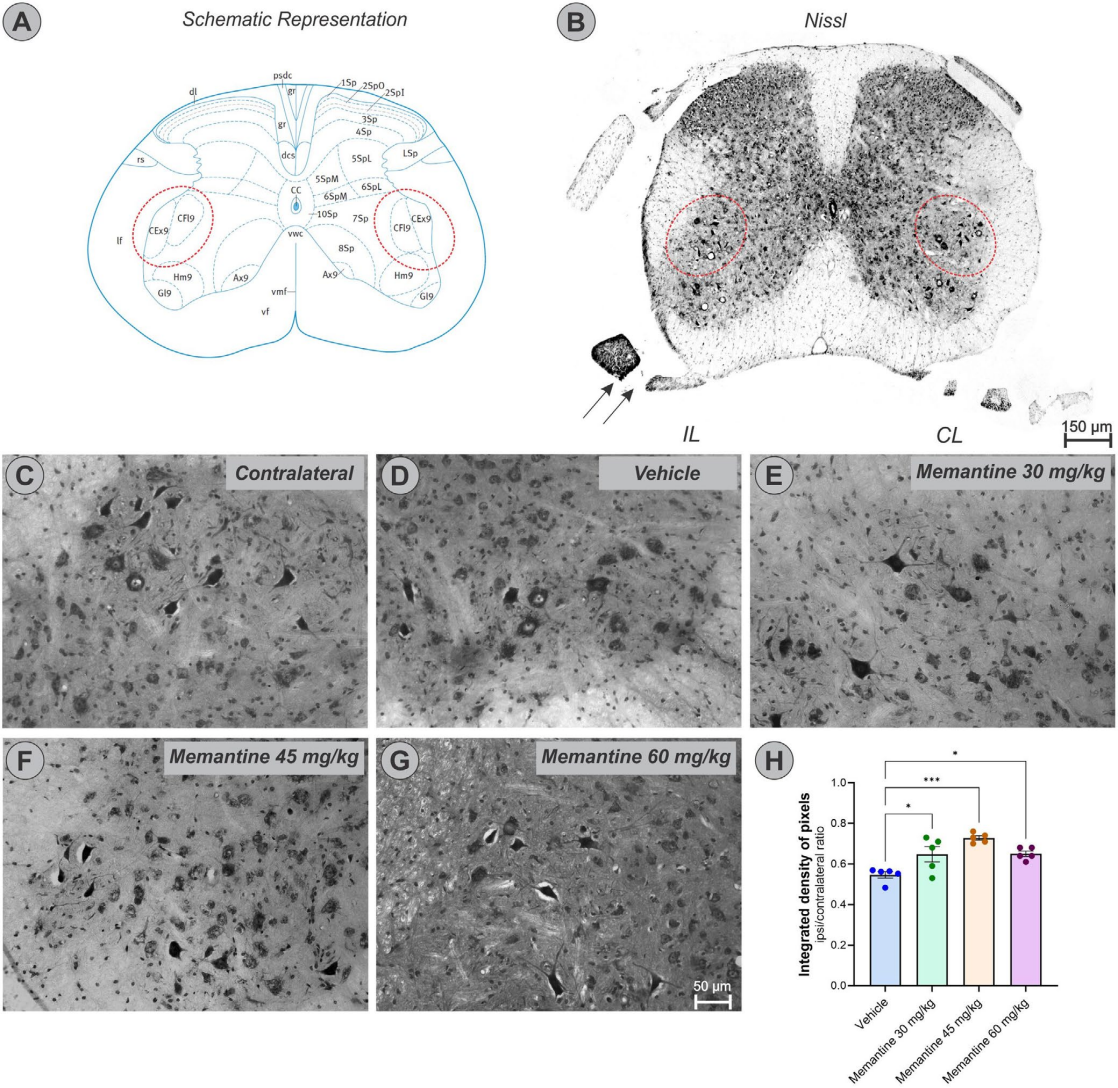
Motoneuron survival 28 days after ventral root crush. Representations of cross‐sections of the spinal cord, at L5 level, showing in (A), a diagram of the distribution of Rexed laminae in the gray matter of the spinal cord, highlighting (red dotted circles) the dorsolateral nuclei of lamina IX, of particular interest in the study (Image credit: Allen Spinal Cord Reference Atlas ‐ https://mousespinal.brain‐map.org/). (B) Panoramic image taken after staining with toluidine blue, highlighting the region of interest on the ipsilateral (IL) and contralateral (CL) sides. Arrows indicate the region where the ventral root was crushed. Scale bar = 150 μm. (C) represents the contralateral motoneuron pool, followed by representative images of the ipsilateral motoneuron pool of (D) Vehicle, (E) Memantine 30 mg/kg, (F), Memantine 45 mg/kg and (G) Memantine 60 mg/kg experimental groups (*n* = 5 mice per group). (H) Quantification of motoneuron survival through the ipsi/contralateral ratio (mean ± SEM; error bars represent SEM). One‐way ANOVA followed by Tukey's post‐test; *p < 0.05; ***p < 0.001.

Transverse sections of the lumbar spinal cord (L4–L6) stained with toluidine blue enabled identification and quantification of spinal motoneurons. Neuronal identification was based on both somatic size (approximately 35 μm in diameter) and localization within the dorsolateral nuclei of the lamina IX of Rexed (Figure 2).

Motoneuron counts were performed bilaterally, on both the ipsilateral (lesioned) and contralateral (uninjured) sides. No significant differences were observed among groups on the contralateral side (Figure S2; one‐way ANOVA *F*_(3,15)_ = 1.88, *p* = 0.1762). In contrast, the ipsilateral side showed a significant reduction in motoneuron number in all experimental groups. To correct inter‐individual variability, ipsilateral‐to‐contralateral (IL/CL) ratios were calculated as an index of neuronal survival 28 days post‐injury (Figure 2). A one‐way ANOVA revealed a significant effect of treatment on IL/CL ratios (*F*_(3,15)_ = 10.30, *p* = 0.00006). Vehicle‐treated animals exhibited approximately 46% motoneuron loss (IL/CL ratio = 0.54 ± 0.02), whereas memantine treatment conferred significant neuroprotection at all doses tested: 30 mg/kg (0.65 ± 0.03), 45 mg/kg (0.73 ± 0.01), and 60 mg/kg (0.65 ± 0.01). Post hoc comparisons confirmed significant differences between the vehicle group and all memantine‐treated groups (*p < 0.05 for 30 mg/kg, ***p < 0.001 for 45 mg/kg, and *p < 0.05 for 60 mg/kg). No significant differences were observed among the memantine doses themselves.

### 3.2 Influence of Memantine Treatment on Reactive Gliosis

#### 3.2.1 Microglia

**FIGURE 3:**
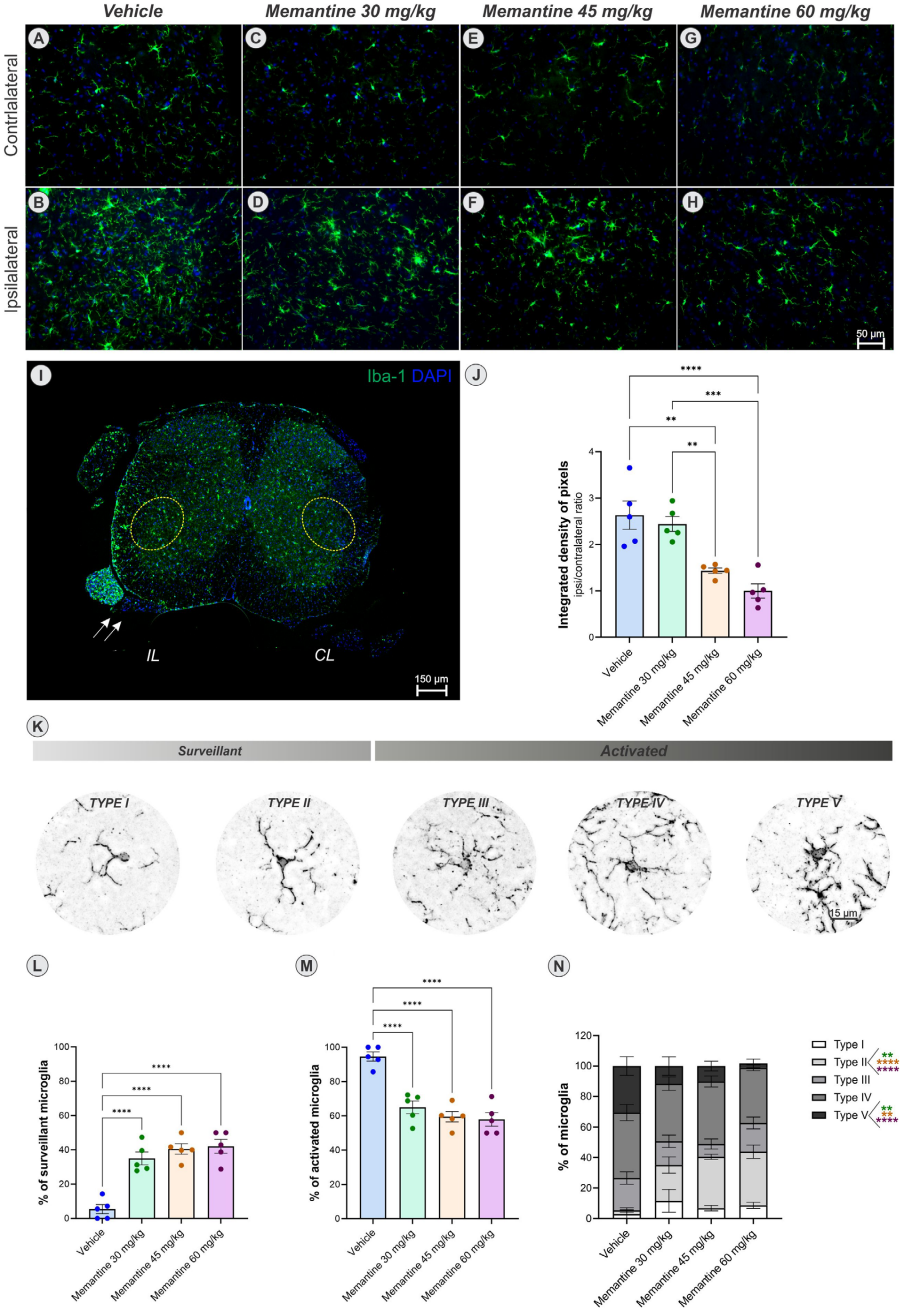
Microglial reaction assessed by Iba‐1 immunostaining (green), counterstained with DAPI (blue). (A, B) represent the contralateral and ipsilateral sides of the vehicle group, respectively. (C, D) memantine 30 mg/kg group. (E, F) memantine 45 mg/kg group, and (G, H) memantine 60 mg/kg group (*n* = 5 mice per group). (I) Panoramic view of the spinal cord cross section, evidencing the IL and CL motoneuron pools within the yellow dotted circles and the crushed ventral root (arrow). (J) Graph of the quantification of the integrated density of pixels (ipsi/contralateral ratio), showing that there was no significant difference in the dose of 30 mg/kg in relation to the vehicle (*p* = 0.88), but there was a significant reduction in the microglial response in the anterior column of the spinal cord after treatment with memantine at doses of 45 mg/kg (**p < 0.01) and 60 mg/kg (***p < 0.001). (K) illustrates the morphological types of microglia classified as surveillant or activated. (L, M) show the proportion of surveillant and activated microglia, respectively, across the experimental groups. Memantine treatment significantly increased the percentage of surveillant microglia and correspondingly reduced the percentage of activated microglia (one‐way ANOVA followed by Tukey's post‐test, ****p < 0.0001). (N) depicts the distribution of microglial subtypes on the ipsilateral sides of all experimental groups (two‐way ANOVA followed by Tukey's post test) (mean ± SEM; error bars represent SEM).

In the present study, following ventral root crush injury, we observed a classical pattern of microglial activation, with a marked increase in microglial density on the ipsilateral side, particularly of cells exhibiting the activated morphology (Figure 3). A one‐way ANOVA revealed a significant effect of treatment on integrated density of pixels (*F*_(3,16)_ = 16.97, *p* < 0.0001). Post hoc comparisons indicate a significant reduction in microglial density at doses of 45 mg/kg (**p < 0.01) and 60 mg/kg (***p < 0.001) compared to both the vehicle and the 30 mg/kg groups.

Moreover, the morphological analysis on the ipsilateral side showed that the majority of Iba‐1 positive cells displayed an activated phenotype (95%–57%). A one‐way ANOVA revealed a significant effect of treatment on the proportion of activated microglia (*F*_(3,16)_ = 25.84, *p* < 0.0001). Post hoc comparisons confirmed a marked reduction in activated microglia, accompanied by an increase in surveillant microglia, in all memantine‐treated groups compared to the Vehicle (****p < 0.0001).

A more detailed characterization of microglial subtypes using a two‐way ANOVA showed no significant main effect of treatment (*F*_(3,79)_ = 0.008, *p* > 0.05), indicating that when data from all microglia types were analyzed together the treatment groups did not differ overall. However, post hoc multiple comparisons revealed subtype‐specific effects: memantine increased the proportion of Type II microglia at 30 mg/kg (**p < 0.01), 45 and 60 mg/kg (****p < 0.0001), while reducing Type V microglia at 30, 45 (**p < 0.01) and 60 mg/kg (****p < 0.0001). These findings indicate that treatment effects were dependent on microglial subtype.

On the contralateral side, most microglia were classified as surveillant (82%–85%). Memantine at 60 mg/kg reduced the proportion of activated microglia and increased the proportion of surveillant microglia compared with the Vehicle. Post hoc analyses indicated that this effect was primarily driven by changes in Type II microglia. Full statistical details are provided in Figure S3.

#### 3.2.2 Astrocytes

**FIGURE 4:**
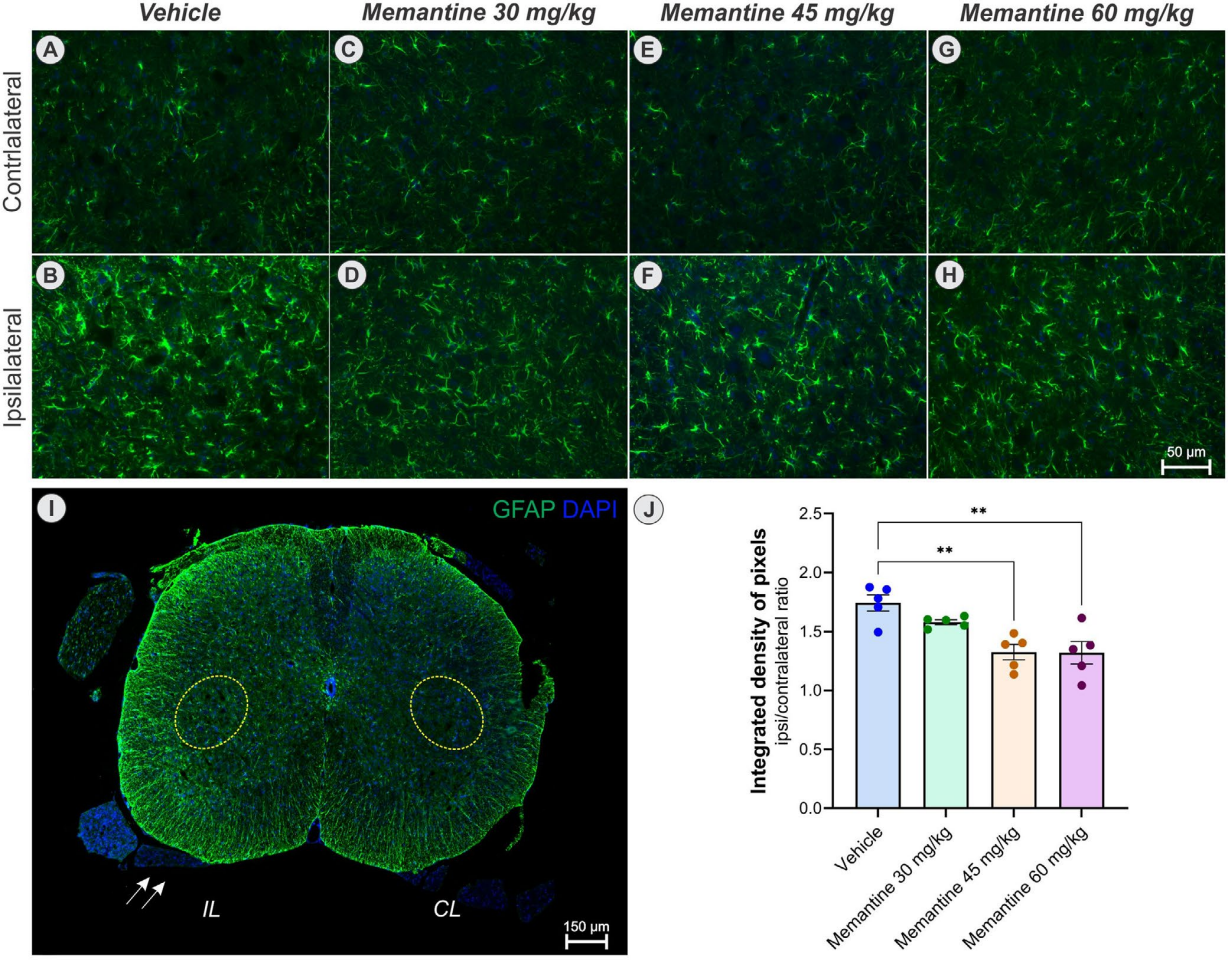
Anti‐GFAP immunohistochemistry (green), counterstained with DAPI (blue) in the different experimental groups. (A, B) represent the contralateral and ipsilateral sides of the vehicle group, respectively. (C, D) Memantine 30 mg/kg group. (E, F) Memantine 45 mg/kg group, and (G, H) Memantine 60 mg/kg group (*n* = 5 mice per group). (I) Panoramic view of the spinal cord cross section, evidencing the IL and CL motoneuron pools within the yellow dotted circles and the crushed ventral root (arrow). (J) Graph of the quantification of the integrated density of pixels (ipsi/contralateral ratio), evidencing the reduction of reactive astrogliosis in all groups treated with memantine in relation to the vehicle group. Note that there was no significant difference between the treated groups (mean ± SEM; error bars represent SEM). One‐way ANOVA followed by Tukey's post‐test. **p < 0.01.

Immunohistochemical analysis using anti‐GFAP staining (Figure 4) clearly demonstrates an increase in reactive astrogliosis on the ipsilateral side compared to the contralateral side. Notably, treatment with memantine resulted in a significant reduction in astrogliosis (one‐way ANOVA, *F*_(3,16)_ = 9.27, *p* < 0.001). Post hoc comparisons indicate a significant reduction in the astrogliosis at doses of 45 and 60 mg/kg (**p < 0.01) compared to the vehicle.

### 3.3 Influence of Memantine Treatment on Synaptic Coverage

To investigate the impact of memantine treatment following ventral root crush injury on both total and differential synaptic stripping within the motor nucleus of the spinal ventral horn, we analyzed the immunoreactivity for synaptophysin, VGLUT‐1, and GAD65.

**FIGURE 5:**
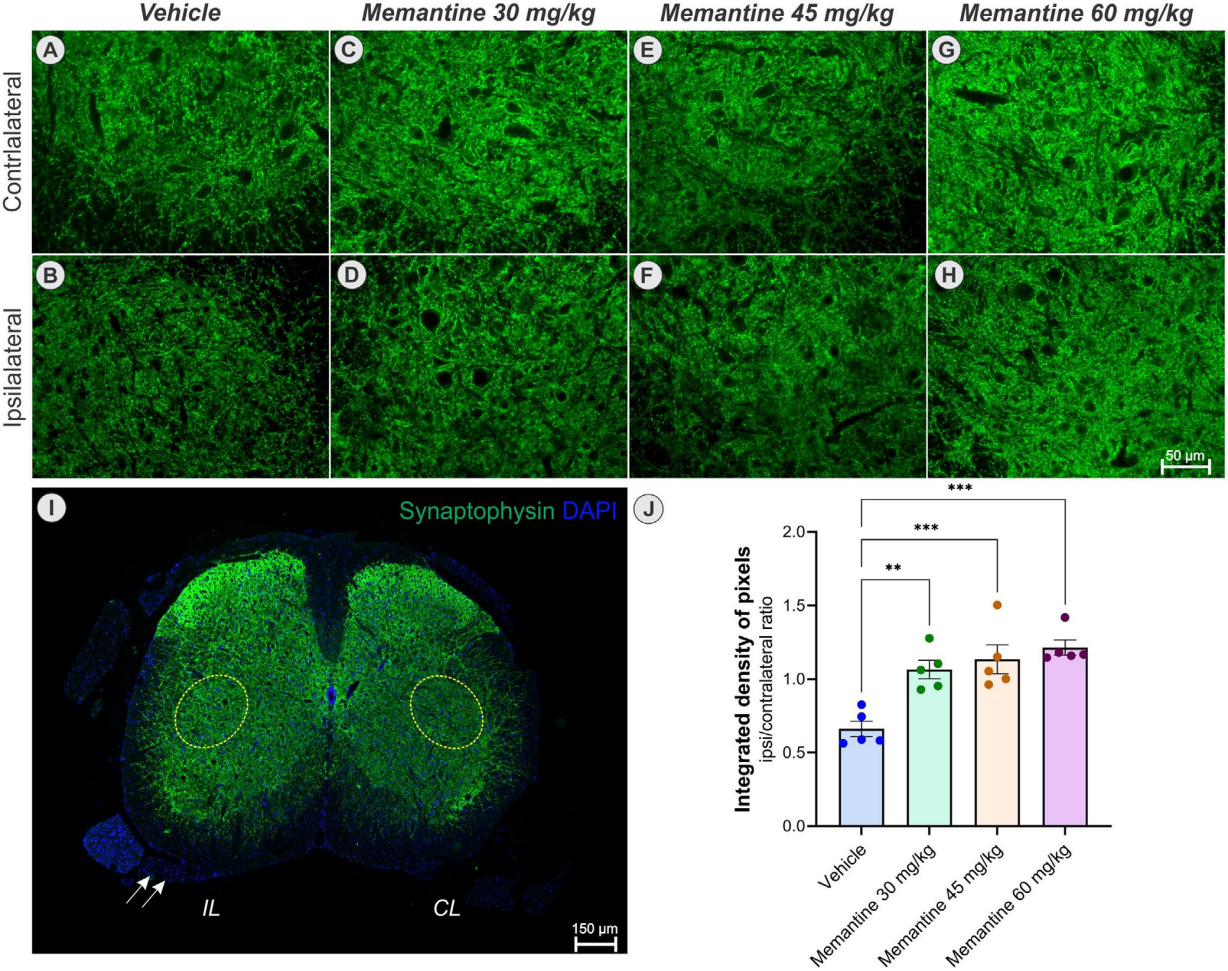
Synaptic coverage, measured by anti‐synaptophysin immunolabeling (green), counterstained with DAPI (blue), in the different experimental groups. (A, B) represent the contralateral and ipsilateral sides of the vehicle group, respectively. (C, D) Memantine 30 mg/kg group. (E, F) Memantine 45 mg/kg group, and (G, H) Memantine 60 mg/kg group (*n* = 5 mice per group). (I) Panoramic view of the spinal cord cross section, evidencing the IL and CL motoneuron pools within the yellow dotted circles and the crushed ventral root (arrow). (J) Graph of the quantification of the integrated density of pixels (ipsi/contralateral ratio), evidencing a significant preservation of synaptic coverage at all doses studied, with no significant difference between them (mean ± SEM; error bars represent SEM). One‐way ANOVA followed by Tukey's post‐test. **p < 0.01, ***p < 0.001.

Immunolabeling for synaptophysin (Figure 5) revealed a marked reduction in synaptic inputs to α‐motoneurons on the ipsilateral side relative to the contralateral side in the vehicle group. In contrast, memantine‐treated groups, across all doses, exhibited comparable synaptophysin labeling between ipsilateral and contralateral sides (one‐way ANOVA, *F*_(3,16)_ = 12,87, *p* < 0.001). Notably, axotomized motoneurons from memantine‐treated animals displayed attenuated synaptic loss, or even a potential increase in synaptic terminals juxtaposed to the soma, compared to vehicle controls, suggesting a reduction in synaptic stripping (**p < 0.01 for the 30 mg/kg group; ***p < 0.001 for the 45 and 60 mg/kg groups). No significant differences were observed among the doses.

**FIGURE 6:**
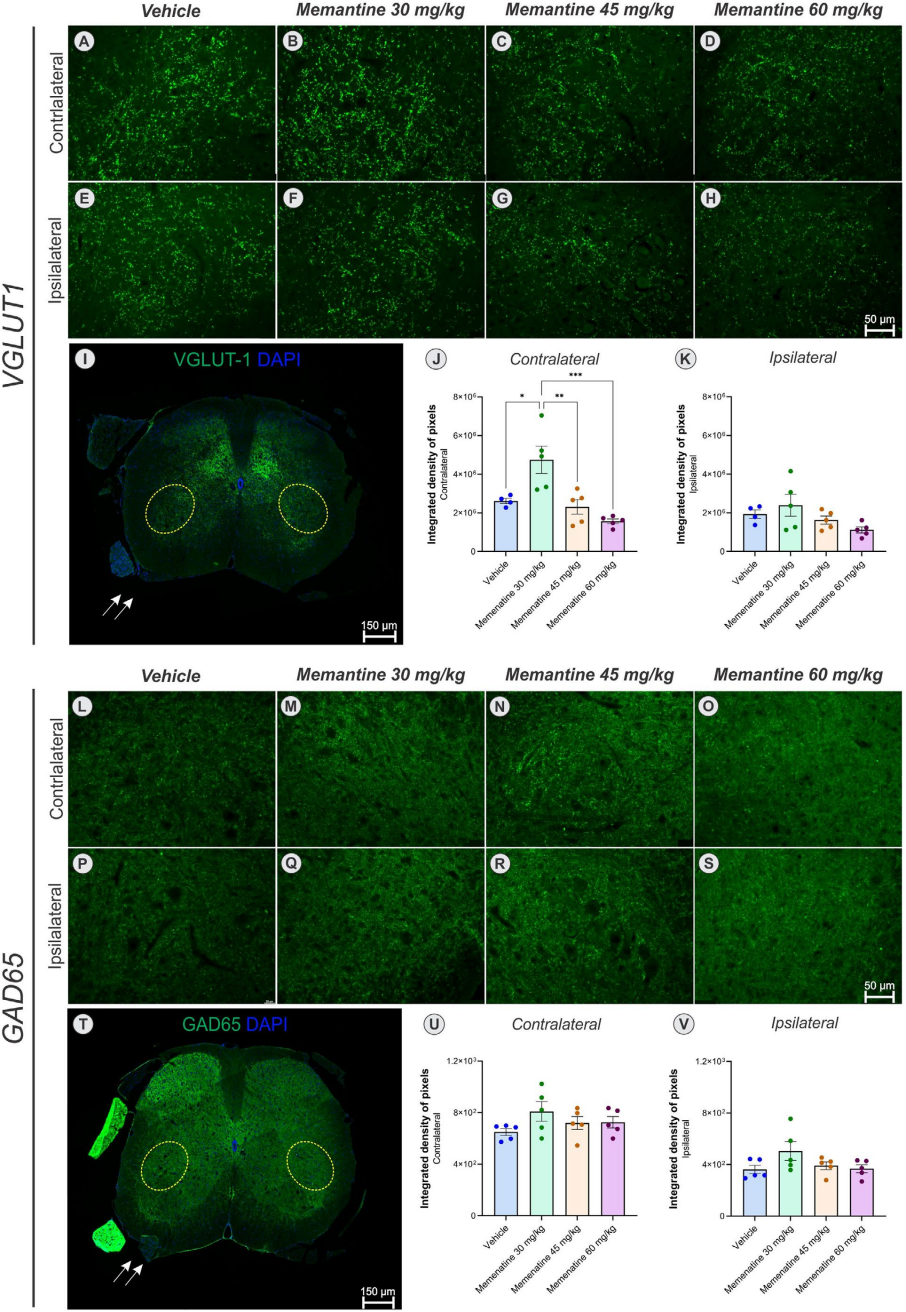
VGLUT‐1 (glutamatergic terminals, green) and GAD65 (GABAergic terminals, green) immunoreactivity, counterstained with DAPI (blue) in the contralateral (CL) and ipsilateral (IL) motoneuron pools across experimental groups (*n* = 5 mice/group; one VGLUT‐1 outlier excluded via ROUT method). (A–H) VGLUT‐1 immunostaining in vehicle and memantine 30, 45, and 60 mg/kg (CL and IL). (I) Panoramic spinal cord cross‐section (VGLUT‐1 and DAPI), showing the IL and CL motoneuron pools (yellow dotted circles), and crushed ventral root (arrow). (J, K) VGLUT‐1 integrated density of pixels quantification in the contralateral and ipsilateral sides, respectively (***p < 0.001, **p < 0.01, *p < 0.05; one‐way ANOVA and Tukey post test; mean ± SEM). (L–S) GAD65 immunostaining in vehicle and memantine 30, 45, and 60 mg/kg (CL and IL). (T) Panoramic spinal cord cross‐section (GAD65 and DAPI), showing the IL and CL motoneuron pools (yellow dotted circles), and crushed ventral root (arrow). (U, V) GAD65 integrated density of pixels quantification in the contralateral and ipsilateral sides, respectively (One‐way ANOVA followed by Tukey's post‐test; mean ± SEM).

To further characterize the preserved synaptic inputs, we quantified immunoreactivity for VGLUT‐1 (a marker of glutamatergic terminals) and GAD65 (a marker of GABAergic terminals) on both contralateral and ipsilateral sides (Figure 6).

Concerning VGLUT‐1, a one‐way ANOVA revealed a significant effect of treatment on the contralateral side (*F*_(3,15)_ = 10.56, *p* < 0.001). Post hoc analysis indicated that the integrated density of pixels was significantly higher in the memantine 30 mg/kg group compared to the vehicle (*p* < 0.05), memantine 45 mg/kg (*p* < 0.01), and memantine 60 mg/kg (*p* < 0.001) groups; one data point was excluded as an outlier using the ROUT method. No significant differences were detected between the experimental groups on the ipsilateral sides (*F*_(3,15)_ = 2.54, *p* = 0.09).

Regarding GAD65 immunoreactivity, a one‐way ANOVA revealed no significant differences among groups on either the contralateral (*F*_(3,16)_ = 1.55, *p* = 0.24) or ipsilateral (*F*_(3,16)_ = 2.19, *p* = 0.13) sides.

### 3.4 Influence of Memantine Treatment on Gene Expression

We further investigated its impact on the expression of key genes involved in neuronal death and glial reactivity using RT‐qPCR 3 and 7 days after lesion. The 45 mg/kg dose was selected for gene expression analysis, as it yielded the most robust neuroprotective effect relative to the vehicle group.

**FIGURE 7:**
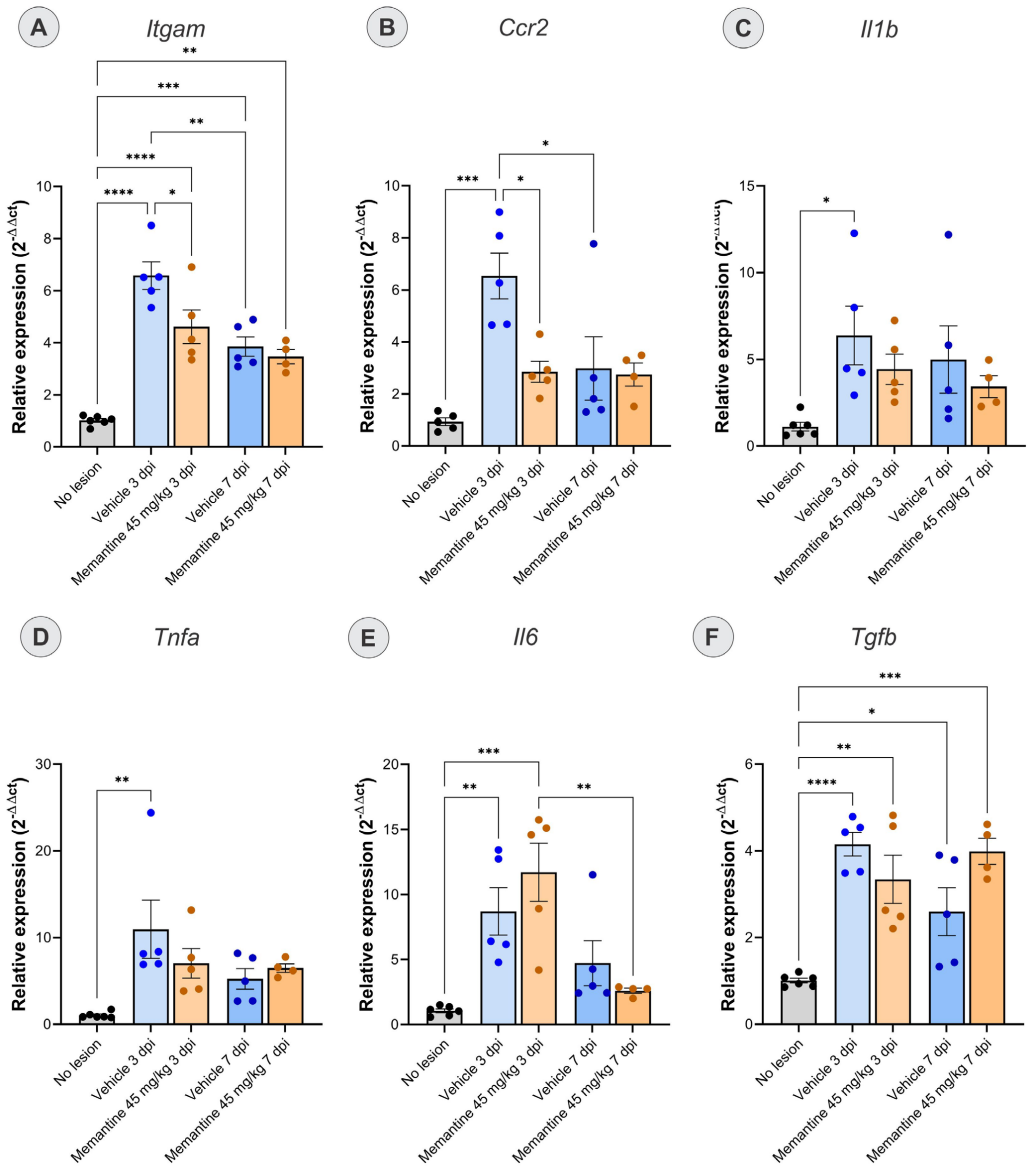
Relative expression of genes involved in monocyte recruitment, and in the inflammatory response in the different experimental groups (*n* = 4/5 mice per group). (A) *Itgam*, (B) *Ccr2*, (C) *Il‐1β*, (D) *Tnf‐α*, (E) *Il‐6*, and (F) *Tgf‐β* (*p < 0.05; **p < 0.01; ***p < 0.001; ****p < 0.0001; one‐way ANOVA followed by Tukey's post‐test; mean ± SEM).

To evaluate the molecular mechanisms underlying memantine's neuroprotective effects, we analyzed two functional gene groups relevant to motoneuron axotomy pathophysiology. The first group comprised markers of the early inflammatory response and monocyte recruitment: *Ccr2, Itgam, Tnf‐α, Tgf‐β, Il‐1β*, and *Il‐6* (Figure 7). TNF‐α, IL‐1β, and IL‐6 are canonical proinflammatory cytokines that are rapidly upregulated in the acute phase of injury. They drive macrophage infiltration and initiate the inflammatory cascade that is characteristic of axotomy (Fregnan et al. 2012; Ma et al. 2024; Nadeau et al. 2011). CCR2 and ITGAM (CD11β) are key mediators of monocyte and macrophage recruitment to the injury site (Rotterman et al. 2019). TGF‐β functions in a dual capacity, initially as a pro‐inflammatory mediator during acute inflammation but subsequently as an anti‐inflammatory regulator that promotes tissue remodeling and repair (Ma et al. 2024).

**FIGURE 8:**
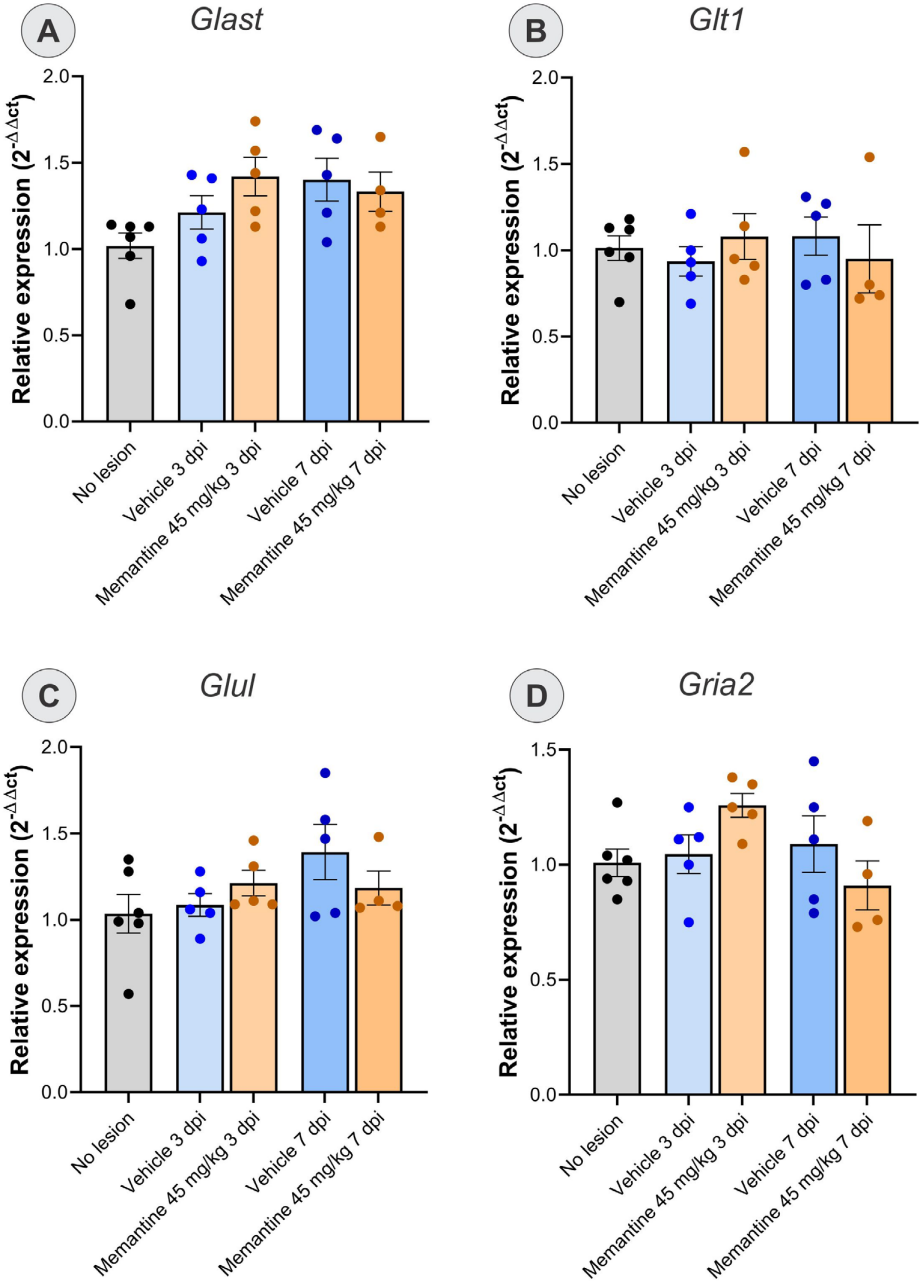
Relative expression of genes involved in glutamatergic signaling in the different experimental groups (*n* = 4/5 mice per group). The graphs demonstrate the analysis of the relative expression of the genes (A) *Glast*, (B) *Glt1*, (C) *Glul*, (D) *Gria2*. There was no significant difference in the expression of these genes after injury or treatment (one‐way ANOVA followed by Tukey's post‐test; mean ± SEM).

The second group comprises genes that regulate glutamatergic signaling and metabolism: *Gria2, Glul, Glt1*, and *Glast* (Figure 8). *Gria2* encodes the AMPA receptor subunit GLUR2, whose calcium impermeability is critical for protecting neurons from excitotoxic calcium overload (Van Damme et al. 2002). GLT‐1 and GLAST are astrocytic glutamate transporters responsible for most of the synaptic glutamate clearance in the CNS (Pajarillo et al. 2019). Glutamine synthase (GLUL) catalyzes the conversion of glutamate to the non‐toxic compound glutamine within astrocytes, representing a critical first‐line defense mechanism against excitotoxicity (Gorovits et al. 1997).

One‐way ANOVA of *Ccr2* relative gene expression revealed a significant effect of treatment (*F*_(4,19)_ = 7.684, *p* = 0.0007). Multiple comparisons analysis indicates an upregulation in the vehicle group at 3 days post‐injury (dpi) compared to the control (*p* < 0.001) and the memantine‐treated (*p* < 0.05) groups. Furthermore, the increase observed in the vehicle group at 3 dpi was reduced by the 7th day after lesion (*p* < 0.05). One‐way ANOVA of *Itgam* relative gene expression revealed a significant treatment effect (*F*_(4,20)_ = 25.11, *p* < 0.0001). Multiple comparisons analysis showed an upregulation in the vehicle group at 3 and 7 dpi (*p* < 0.001) compared to the control group. This increase was significantly lower in the memantine group at 3 dpi compared with the vehicle group (*p* < 0.05).

Analysis of additional inflammatory genes revealed distinct temporal patterns. *Il‐1β* and *Tnf‐α* showed significant differences in the vehicle group compared to the control group at 3 dpi (*F*_(4,20)_ = 2.767, *p* = 0.05 for *Il‐1β* and *F*_(4,20)_ = 4.418, *p* = 0.01 for *Tnf‐α*). In contrast, *Tgf‐β* showed significant differences among the experimental groups (*F*_(4,20)_ = 11.90, *p* < 0.0001). Multiple comparisons showed increased expression in vehicle and memantine groups at 3 (*p* < 0.0001 and *p* < 0.01, respectively) and at 7 dpi (*p* < 0.05 and *p* < 0.001, respectively).

For genes involved in glutamatergic metabolism and signaling (Figure 8), one‐way ANOVA of *Glast* revealed significant differences (*F*_(4,20)_ = 2.871, *p* < 0.05), but the post hoc Tukey test showed no statistically significant intergroup differences. One‐way ANOVA revealed no significant differences for *Glt1, Glul*, and *Gria2* (*Glt1*: *F*_(4,20)_ = 0.3358, *p* = 0.8506; *Glul*: *F*_(4,20)_ = 1.657, *p* = 0.1933; Gria2: *F*_(4,20)_ = 2.075, *p* = 0.1222).

## 4 Discussion

The present study demonstrates that memantine exerts consistent neuroprotective effects after ventral root crush injury, with a clear dose‐dependent profile that peaks at 45 mg/kg. Importantly, neuroprotection was evident across all doses tested, reinforcing the robustness of NMDA receptor antagonism as a therapeutic strategy. This observation aligns with previous reports describing that partial, non‐competitive inhibition of NMDA receptors mitigates excitotoxic neuronal injury while preserving physiological glutamatergic signaling (Lipton 2006; Wu et al. 2009). Further, the presence of extrasynaptic NMDA receptor selectivity is a hallmark of memantine's neuroprotective action (Kuns et al. 2025; Xia et al. 2010). Activation of extrasynaptic NMDA receptors is linked to pro‐death signaling pathways and mitochondrial dysfunction, whereas synaptic NMDA activity supports neuronal survival and neuronal plasticity (Hardingham and Bading 2010). Therefore, this selective inhibition provides a mechanistic basis for memantine's ability to suppress pathological glutamatergic activity while sparing normal physiological transmission, in line with the neuroprotective outcomes observed herein.

The dosages and treatment duration chosen for the present study were based on previous in vivo studies indicating that memantine efficacy is highly dose‐dependent (Dong et al. 2008; Okamoto et al. 2009; Siddharthan et al. 2020; Trotman et al. 2015). Three decreasing doses (60, 45, and 30 mg/kg) were designed to establish a dose–response curve, while minimizing the risk of excitotoxicity, allowing for a thorough evaluation of memantine's neuroprotective potential within the critical 14‐day therapeutic window that matches motoneuron degeneration (Cartarozzi et al. 2025, 2022, 2019). This interval represents a critical period during which interventions aimed at counteracting secondary damage, particularly those related to glutamate‐mediated excitotoxicity, can exert maximal neuroprotective effects. Accordingly, memantine was administered throughout this 14‐day period, and histological analyses were carried out at 28 days post‐injury, a stage when neuronal loss and synaptic remodeling are well consolidated, allowing assessment of whether early pharmacological modulation yields sustained structural and neuroprotective outcomes.

Histopathological analysis revealed robust astroglial and microglial activation ipsilateral to the lesion, consistent with previous reports that identify glial cells as key mediators of neuroinflammation and synaptic remodeling after motoneuron injury (Aldskogius et al. 1999; Cartarozzi et al. 2022; Rotterman et al. 2019; Rotterman and Alvarez 2020). Within this context, glial modulation by memantine becomes particularly relevant. By dampening NMDA receptor–mediated signaling in astrocytes and microglia, memantine not only reduces direct excitotoxic neuronal damage but also suppresses maladaptive glial responses, thereby fostering neuronal survival (Sulkowski et al. 2014; Verkhratsky and Kirchhoff 2007).

In microglia, memantine significantly reduced both integrated density of pixels and the proportion of activated cells, suggesting a qualitative shift toward a surveillant phenotype, associated with lower cytotoxicity potential (Nimmerjahn et al. 2005). The ipsilateral/contralateral ratio of the Iba‐1 immunolabeling further supports this interpretation, indicating a dose‐dependent suppression of microglial activation. These findings align with previous models of neuropathic pain and LPS‐induced neuroinflammation, reinforcing a direct relationship between reduced microglial activation and enhanced neuronal survival (Chen et al. 2019).

RT‐qPCR analysis at 3 and 7 dpi characterized early transcriptional responses, revealing pronounced upregulation of genes involved in monocyte recruitment (*Ccr2, Itgam*) and pro‐inflammatory cytokines (*Tnf‐α* and *Il‐1β*) in vehicle‐treated animals, which memantine effectively suppressed while also modulating *Il‐6* expression. These acute molecular changes temporally precede the glial and structural alterations assessed histologically at 28 dpi. While early gene expression cannot be directly extrapolated to later time points, the present molecular data suggest that memantine modulates the initial inflammatory milieu in a manner compatible with subsequent neuroprotection.

Astrocytes exhibited typical features of reactive astrogliosis (Sofroniew 2014). Giving the established role of the astrocytic NMDA receptor activity in ionic balance and glutathione synthesis (Jimenez‐Blasco et al. 2015), memantine's modulation of these receptors may contribute to improved redox control and ionic homeostasis (Obara‐Michlewska et al. 2015). The attenuation of astrogliosis observed at 28 dpi therefore likely reflects an additional protective effect, consistent with reports describing anti‐inflammatory actions of memantine after CNS injury (Suhs et al. 2016). Importantly, no significant changes were detected in the early expression of glutamate transporter or receptor genes (*Gria2, Glul, Glt1, Glast*), implying that memantine's initial actions rely on post‐transcriptional and signaling mechanisms rather than direct transcriptional regulation of glutamate metabolism.

Another relevant aspect concerns the process of synaptic stripping, which is well described following motoneuron axotomy. Under normal conditions, there is a well‐known 4:1 ratio of inhibitory to excitatory inputs to the alpha spinal motoneurons (Linda et al. 2000). Following injury, however, this ratio changes dramatically due to a consistent drop in inhibitory synapses, which is largely dependent on MHC‐I expression by motoneurons (Oliveira et al. 2004). The resulting predominance of glutamatergic boutons may exacerbate excitotoxic stress and correlate with persistent loss of Ia proprioceptive afferents (Alvarez et al. 2020). As previously discussed, memantine's action on extrasynaptic NMDA receptors may balance neuronal excitability. This ensures the recovery of motoneuron innervation and proper inhibition‐excitation correlation. This is crucial for functional recovery, including activation of spinal reflexes and pattern‐generator circuits.

Our findings of synaptic preservation under reduced glial activation suggest that memantine, by modulating glial responses, may promote neuronal survival and preserve critical spinal connectivity required for functional recovery. Notably, memantine produced a dose‐dependent effect on contralateral VGLUT‐1 expression, with the 30 mg/kg dose increasing immunoreactivity compared to higher doses and vehicle, while ipsilateral VGLUT‐1 and GAD65 on both sides remained equivalent. This contralateral‐selective increase may reflect either preferential extrasynaptic NMDA blockade at lower doses, allowing VGLUT‐1 upregulation, or compensatory VGLUT‐1 expression in response to partial NMDA inhibition, both consistent with dose‐dependent NMDA effects on synaptic plasticity (Lipton 2006; Wu et al. 2009).

Nevertheless, further ultrastructural analysis is necessary to more precisely assess ipsilateral axotomized and contralateral motoneurons coverage and properly evaluate synapses. This includes quantifying excitatory and inhibitory pre‐synaptic boutons. Additionally, it is of interest to correlate VGLUT‐1 expression with the number of vesicles within glutamatergic boutons, as memantine may also interfere with the amount of glutamate released into the synaptic cleft (Lu et al. 2010).

Taken together, these findings indicate that memantine's neuroprotective efficacy arises from a synergistic interplay between its anti‐excitotoxic and anti‐inflammatory properties. By selectively dampening extrasynaptic NMDA receptor activity, mitigating microglial and astroglial activation, repressing pro‐inflammatory gene expression, and preserving synaptic circuitries, memantine creates a permissive microenvironment for motoneuron survival. These results expand the translational potential of memantine as a therapeutic intervention in conditions characterized by concurrent excitotoxicity and neuroinflammation. Future work should refine therapeutic timing and explore combinatorial strategies with rehabilitation to optimize long‐term functional recovery.

## 5 Conclusion

Memantine provides dose‐dependent neuroprotection after ventral root crush, primarily through the modulation of glial reactivity and suppression of acute inflammatory responses. By reducing microglial activation, attenuating astrogliosis, and limiting cytokine upregulation, memantine promotes a neuroprotective environment that enhances motoneuron survival. These findings highlight its translational potential as a therapeutic strategy for disorders driven by excitotoxicity and neuroinflammation.

## Author Contributions

**Arthur Ventura Martins Leão:** methodology, investigation, writing – original draft. **Gabriel Gaspar Bíscaro:** methodology, investigation, writing – original draft. **Alexandre Leite Rodrigues de Olivera:** conceptualization, methodology, funding acquisition, writing – review and editing, supervision. **Luciana Politti Cartarozzi:** conceptualization, methodology, writing – review and editing, supervision, funding acquisition.

## Funding

This work was supported by the São Paulo Research Foundation—FAPESP (Grants 2021/05180‐3, 2022/06609‐6, 2023/16415‐7, 2023/02615‐4, and 2024/01736‐5) and the National Council for Scientific and Technological Development—CNPq (Grant 303050/2021‐7).

## Conflicts of Interest

The authors declare no conflicts of interest.

## Supporting information


**Figure S1:** jnc70429‐sup‐0001‐Supinfo01.docx.

## Data Availability

The data that support the findings of this study are available from the corresponding author upon reasonable request.
