# Supplementary material for: Targeting Glutamate Excitotoxicity With Memantine Modulates Glial Response and Protects Motoneurons After Spinal Root Lesion
Source: J Neurochem. 2026 Apr 8;170:e70429. doi: 10.1111/jnc.70429 (PMC13060370; doi:10.1111/jnc.70429)
Supplement: Supplementary file 1 — Figure S1: jnc70429‐sup‐0001‐Supinfo01.docx. [file JNC-170-e70429-s001.docx]

# **Targeting Glutamate Excitotoxicity with Memantine Modulates Glial Response and Protects Motoneurons After Spinal Root Lesion**

Arthur Ventura Martins Leão^1§^, Gabriel Gaspar Bíscaro^1§^, Alexandre Leite Rodrigues de Olivera^1^, Luciana Politti Cartarozzi^1*^

^1^Laboratory of Nerve Regeneration, Institute of Biology – University of Campinas. Campinas, São Paulo/Brazil.

***
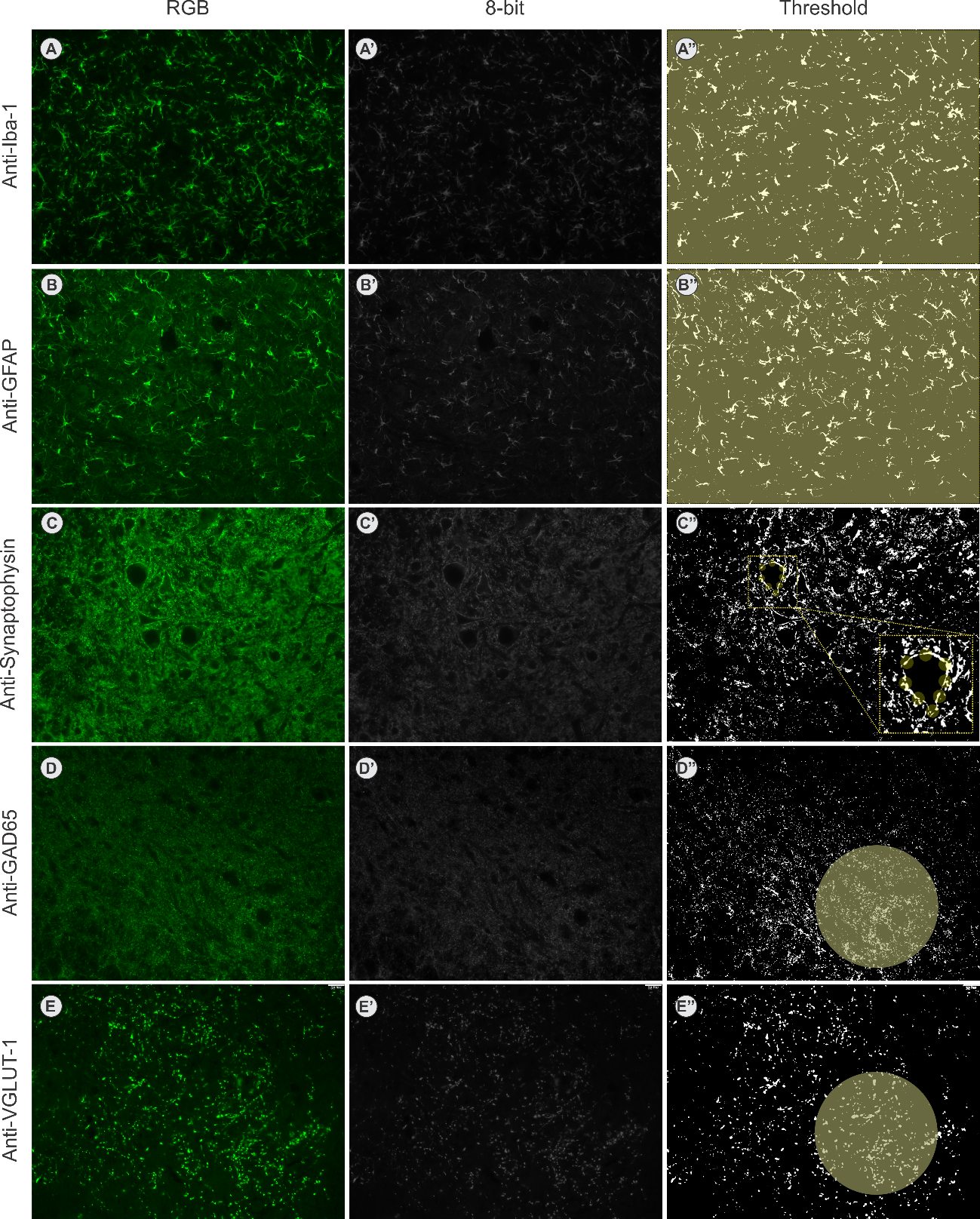
***

***Suppl. Fig. 1 -*** Workflow for quantification of integrated pixel density for each antibody used, showing the images obtained in RGB (A - E); in 8-bit (A’ - E’) and the images after definition of the threshold (threshold, A’’ - E’’). Yellow areas indicate the regions sampled for quantification of integrated density of pixels: the whole image for Iba-1 and GFAP (A’’, and B’’); eight equidistant points around the cell body of axotomized motoneurons, for the anti-synaptophysin (C’’), and a circular region over the lateral motor nucleus (shown as yellow circles in panels D’’ and E’’), for GAD65 and VGLUT-1.


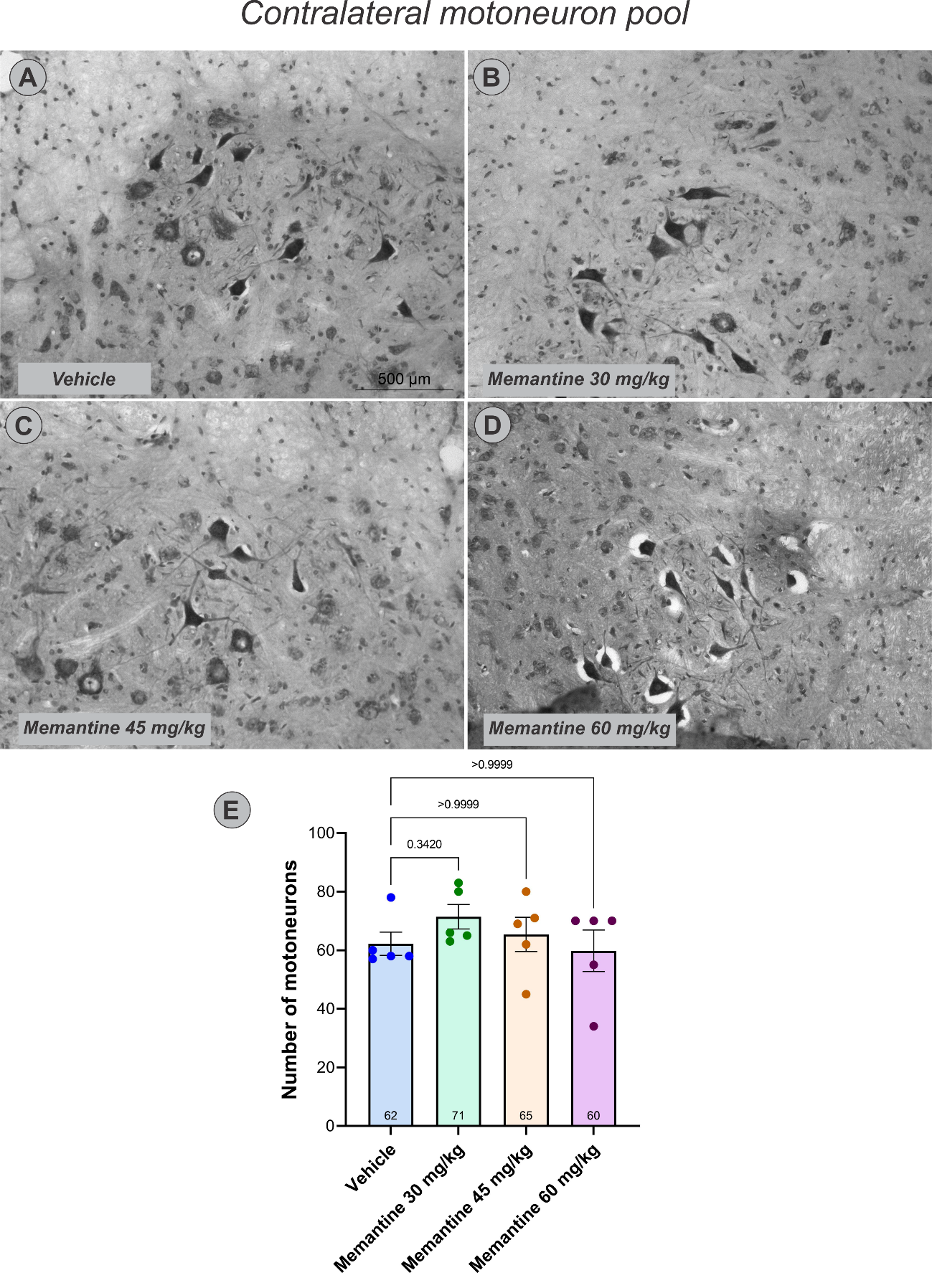


**Suppl. Fig. 2 –** Contralateral motoneuron pool in all experimental groups. Representative images of the contralateral dorsolateral motoneuron pool in the Vehicle (A), Memantine 30 mg/kg, (C) 45 mg/kg, and (D) 60 mg/kg (n = 5 mice per group). Analysis of the total number of motoneurons in the contralateral pool (E) did not show significant differences among experimental groups (mean ± SEM; error bars represent SEM. One-way ANOVA and Tukey’s post test).


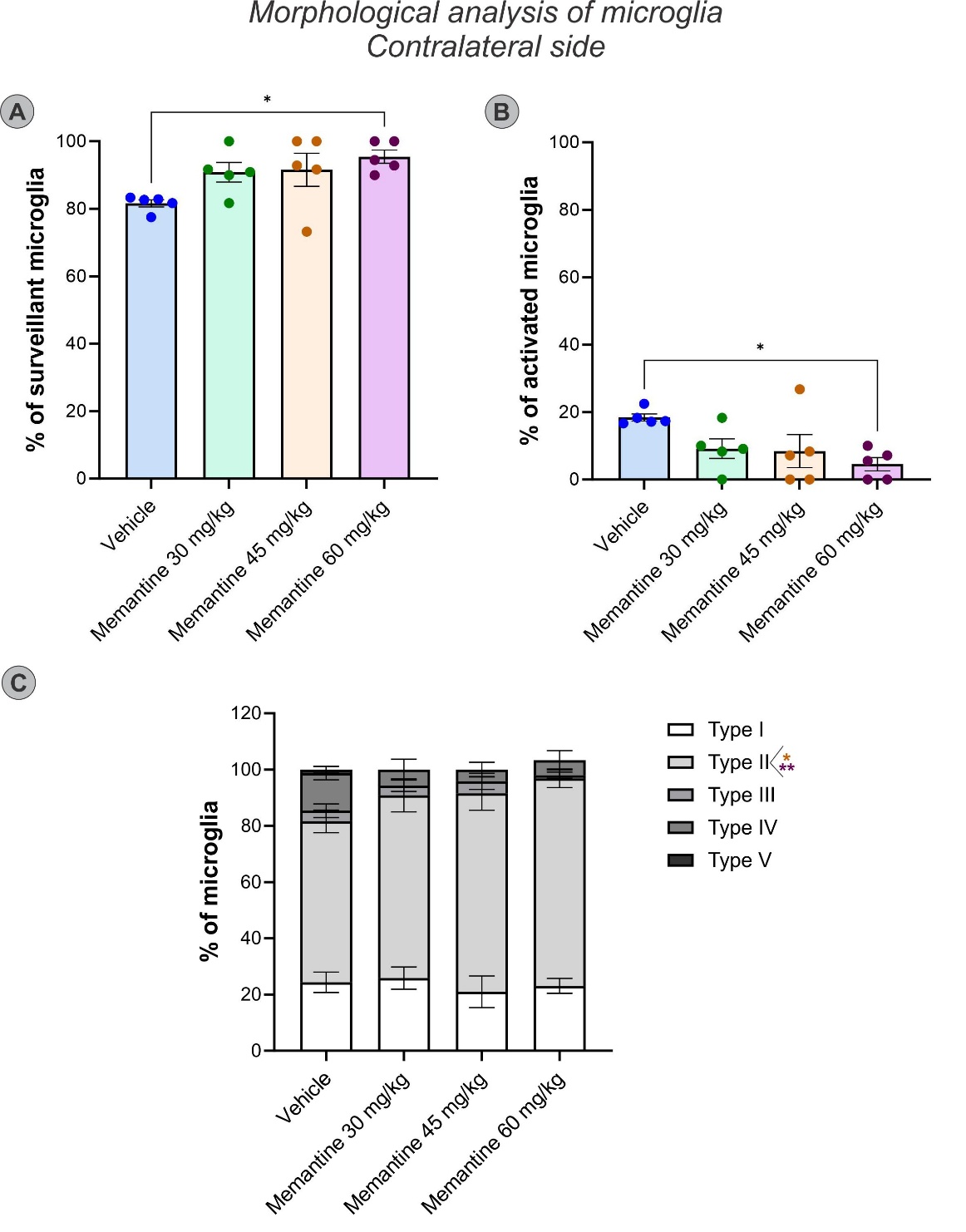


**Suppl. Fig. 3 –** Quantification of microglial phenotypes in the contralateral side. The majority of Iba‑1–positive cells were classified as surveillant microglia (82–95%) across all groups. A one‑way ANOVA revealed a significant effect of treatment on the proportion of (A) surveillant (B) activated microglia (F_(3,16)_ = 3.67, p = 0.03), with Tukey post‑hoc test indicating a reduction in activated microglia and a corresponding increase in surveillant microglia in the 60 mg/kg memantine group compared with the Vehicle (*p<0.05). (C) A two‑way ANOVA revealed no significant interaction between treatment and microglial subtype (F_(1,736)_ = 0.081, p > 0.05) and no main effect of treatment (F_(1,736)_ = 0.012, p>0.05). However, there was a significant main effect of microglial subtype (F_(1,736)_ = 91.46, p<0.0001). Post‑hoc Tukey comparisons showed that memantine produced subtype‑specific effects at higher doses. Compared with the Vehicle group, memantine at 45 mg/kg significantly reduced the proportion of activated microglia (*p<0.05), and memantine at 60 mg/kg produced an even greater reduction (**p<0.01). These changes were primarily driven by an increased frequency of Type II microglia. Data are presented as mean ± SEM.
